# On the Use of Turpentine

**Published:** 1853-12

**Authors:** Samuel Thompson

**Affiliations:** Albion, Ill.


From the Transactions of the Illinois State Medical Society.

*On the Uses of Turpentine.* By Samuel Thompson, M. D., Albion, Ill.

In presenting this paper to the Society, my object is to call the attention of the profession to the therapeutic application of an article well known to all and much used by some, but which, as far as I am informed, has hitherto received but occasional notice, and been applied to its most important uses by only a few individuals.

In my own neighborhood, and among most of my professional friends, it is only a few years since even its use in combination with castor oil, when administered as a purgative, was looked upon with astonishment and distrust; and I well remember the difficulty I had to induce my patients to swallow what they had never known to be prescribed by my predecessors.

Hitherto I believe turpentine has in most cases been prescribed empirically, each individual prescribing it, applying, if he had a turn for such reasoning, (theorizing it will perhaps be called,) his own explanation of its modus operandi. Now it is the fashion to speak very slightingly, and very philosophically of attempts to ascertain the mode of action of medicines; but I must confess, that I belong to the few who consider that it is *not* visionary to seek to know how medicines produce their effects, and that just in proportion as we arrive at that knowledge, will be the scfentific accuracy with which we shall prescribe them. I shall in this paper, therefore, endeavor to present what time and circumstances have enabled me to collect of the opinions and the experience of others as to the uses of turpentine in disease, adding the results of its use in my own practice. And I think it will thence be seen, that the cases in which it has been successfully applied, will point to its modus operandi, and at the same time mark out a wide range of affections, involving similar functional or structural derangements, though in diseases bearing different names, and to which very different organs are subject, and in which cases we shall find it a useful agent and trusty friend.

In looking over systematic works and periodicals for what had been written on this subject, I find that the diseases in which turpentine has been recommended, are :

Iritis, by five authors.*

Typhoid pneumonia, by one.†

Amaurosis, by one.‡

Tania, by one.††

Hemorrhage from mucous surfaces, and also in cases of purpura, by six.‡‡

Dysentery, by four.**

Colic, by one.¶

Congested and distended state of rectum, by one.¶¶

Ulcers, by two.***

See also Wood's Practice of Medicine, for its uses in typhoid fever.

In my own practice I find I have notes of about thirty cases, in which I have prescribed it; namely, in dysentery—nine cases, one was accompanied with hæmatemæsis; in diarrhœa, bloody—three cases; in diarrhœa, white—one case; in typhoid fever, with intestinal hemorrhage—three cases. In one case the patient died, but she had been half murdered by a quack before I saw her, and even then, from the carelessness of the nurses combined with the obstinacy of the patient, who was violently delirious, it was only partially administered; in colic, one case—the patient was a toper and a tobacco chewer; in pneumonia, secondary on measles, where the symptoms rendered the use of mercury objectionable. In the spring of 1852 prescribed it in about twelve cases.

The first of these recorded cases in which I have used it, bears

---

*No. 1, Braithwaite, Jan. 1840, p. 87—Arnott & Foote.
14, Braithwaite, July, 1847, p. 268—Langier.
12, do. July, 1845, p. 236—Graves.
Wharton Jones Ophthalmic Surgery.

†No. 2, Braithwaite, July, 1840, p. 58—Martin.

‡No. 5, " Jan., 1842, p. 133—Hocken.

††No. 6, " July, 1842, p. 64—Bellingham.

‡‡No. 7, " Jan., 1843, p. 87—Willshere & Copland.
12, " July, 1845, p. 132—Neligan.
19, " Jan., 1849, p. 143—J. P. Vincent.
American Jour. Med. Sciences, 1850, No. 39, p. 198—T. Smith, John Hunter.

**Trans. Am. Med. Association, 1851, p. 455—Dranghon.
Am. Journal Med. Sciences, 1851, p. 366 and 367—Shattuck.
St. Louis Med. Jour., May, 1852, p. 209—John Long.
Western Jour. Med., July, 1822—Durrett.

¶Am. Jour. Med. Sciences, July, 1851, p. 79—Parker.

¶¶No. 15, Braithwaite, Jan., 1847, p. 115—Dick.

***No. 14, " July, 1847, p. 321—Hancock & Heister.

date 24th July, 1851, and, though I do not now remember upon whose suggestion I was first led to use it in the bold way I have since done, I suspect it was the paper of Dr. Hughes Willshire, in the 12th number of Braithwaite's Retrospect, on its uses in purpura, and wherein he mentions Dr. Copland's view of its modus operandi, to be absorbed into the blood, and its thence producing an astringent effect upon the capillary vessels, that first drew my attention especially to it, as likely to fill a void in therapeutics which I had long felt. The usual quantity which I have administered to an adult in either dysentery or hemorrhagia, has been a common tea-spoonful with from four to six drops of tinct. opium every two or three hours, and given in either milk, mucilage or coffee, and for younger persons in proportion. In several cases, chiefly in those frightful hemorrhages occurring in the latter stages of typhoid fever, its use has been continued for two or three days, or even longer, gradually making the intervals between the doses wider as the flow of blood decreased. In one nearly fatal case its use was combined with the exhibition of injections into the bowels by means of an œsophagus tube, in the first instance using acetate of lead, tinct. of opium and water, and afterwards, that proving inefficient, giving infusion of matico with tinct. of opium, after which the hemorrhage ceased, but the patient was almost in articulo mortis. Two or three days after there was a slight return of the sanguinous discharge, which again stopped upon administering the turpentine. I gave the turpentine in combination with nitric ether also in one case, during the collapse stage of cholera, though only one dose could be got down; at first it appeared to cause some distress, but in a few minutes the patient seemed much better, and spoke clearly for the first and only time after I saw her, but she was quite cold and pulseless everywhere, except on the scalp and epigastrium, and sunk in a few hours.

In July last I administered it in one case to a child, 11 months old, suffering from choleric diarrhœa, which in the first attack had evidently been of an intermittent type, and had been relieved by quinine, &c., but the little patient had subsequently been allowed to eat uncooked green peas, which occasioned a violent relapse.—In this case ten drops of turpentine with one to three drops of laudanum were given every three hours, (the child's skin was quite cold), but it sunk in a few hours from the continued passive discharge of bloody water.

Among the nine cases of dysentery above referred to, one was dying when I first saw it; in nearly every other case the symptoms were relieved in from a few minutes to a few hours; indeed, so prompt was the relief from the tormina and tenesmus, that after the first, the patients were in general very ready to take the medicine, to which at first they felt an almost insuperable aversion.

One of the cases of bloody diarrhœa accompanied a severe attack of remittent fever and was immediately relieved by the turpentine, etc. In another case the attack was distinctly of that adynamic type, of which so many cases occurred during the last cholera epidemic; it also was promptly relieved The other case referred to was a bloody diarrhœa that supervened on an attack of milksickness, in an individual previously in a low state of health, and which for a time threatened to finish what the milksickness had left undone; but it was promptly and effectually relieved by the turpentine.

In the early part of the spring of 1852, measles was epidemic, especially in the regions bordering on the streams; the weather was cold and wet, and, though the robeolous symptoms themselves were generally easily managed, several cases of pneumonia, from exposure, accompanied by a very troublesome diarrhœa or a dysentery occurred, and these same conditions, as the consequence of exposure during *convalescence*, were also very frequent. After resisting the most assiduous use of the usual remedies indeed, the very circumstance of diarrhœa accompanying a pneumonitis, greatly curtailed the usual list of agents, In these cases I found valuable aid from turpentine, generally in combination with spts. ether nit. and tinct. opii. Now I am by no means inculcating the idea, that turpentine is in these cases the specific; for, in fact, I believe before we can find specific remedies, we must so contrive, that diseases shall always have specific grades, and operate always on similar constitutions, and after similar predisposing causes. But I submit, that if we look over the cases referred to in my practice, (I have not copied the details on account of room), and in like manner analyze the cases in our periodical and systematic works, to which I have referred, it will be seen, that in all these cases the structures involved were much the same, the nature of the lesions essentially similar; let us pass them in a brief review.—Iritis—the iris is a fibrous structure, covered with villi floating in a fluid, and devoid of cuticle, thus approximating in its surface and in many of its conditions to mucous membrane. In typhoid pneu monia, in amaurosis (from congestion or pressure of course only), in ulcers, in hemorrhages from any natural outlet, in dysentery, and the various diseases in which it is reported to have been used, the conditions present are essentially derangements of mucous membranes. Then in all these cases involving lesions of this peculiar structure, a structure unprotected by superincumbent parts, and unsupported by a cuticle, but composed almost of a network of capillary vessels, which, from their peculiar position, are specially liable to congestion, and consequent distension and obstruction, we find good testimony that turpentine has rendered good service. Let us see what the action of other approved remedies suggests in reference to the manner in which these diseases can be relieved. In iritis we trust to mercury, and mercury we know to have a tendency to attenuate the blood, thus fitting it more readily to pass through obstructed vessels, and rendering it less liable to coagulate therein. We believe also that it promotes absorption of matters already deposited, in virtue, no doubt, of some power it exerts on the minute vessels. (Query. Does not the information supplied by the principles of exosmoses and endosmoses suggest, that as a vessel in a state of congestion absorbs but little, whatever causes its contents to be lessened, increases its absorbing power?) In passive hemorrhages and in dysentery there must be a deranged condition of the coats of the bloodvessels,* as well as probably an abnormal state of the blood; and this we must suppose to be the case in most of those ailments involving the mucus membrane.

In hemorrhage we give acetate of lead with a view to its astringent effect. Alum and nitrate of silver are used for the same ends. In dysentery it has been recommended by high authority‡ to give large, aye, even ounce doses of cream of tartar, frequently repeated, or sulphate of magnesia in small doses, at short intervals, and in some seasons I have known both of these courses of treatment eminently suceessful; such cases, I presume, being characterized by active congestion of the mucus membrane of the bowels, and the hydragogue action of these drugs acted as local depletives of the engorged organs, while they exerted also a refrigerating influence on the irritated surface. Others again have trusted to calomel and opium, even to ptyalism, or opium alone in heroic doses; in either of these cases, I doubt not, either treating a different shade of the disease, or acting through a different channel of impressibility perhaps simply lulling the excitement of the system, till nature in the round of her functions could restore the balance of action. But I have tried all these, and at times have found each of them to answer; and then again, in another season, and under another epidemic or endemic condition, have been just as bitterly disappointed in their effects; and this, I doubt not, has been the experience of every observant physician.

---

* Wood's Practice of Medicine, vol. 1, p. 257, remarks: "It is impossible to conceive of a discharge of blood without a morbid state of the vessel."

‡ Stokes—Bell & Stokes, vol. 1, p. 211.

